# Macrophages in Focus: Key Drivers and Therapeutic Opportunities in Diabetic Kidney Disease

**DOI:** 10.7150/ijbs.112737

**Published:** 2025-07-11

**Authors:** Hui Zhao, Jia Guo

**Affiliations:** 1 Department of Nephrology, The First Affiliated Hospital of Zhengzhou University, Zhengzhou, Henan, 450052, China.; 2 Key Laboratory of Precision Diagnosis and Treatment for Chronic Kidney Disease in Henan Province, Zhengzhou, Henan, 450052, China; 3 Tianjian Laboratory of Advanced Biomedical Sciences, Academy of Medical Sciences, Zhengzhou University, Zhengzhou, Henan, 450001, China.

**Keywords:** diabetic kidney disease, macrophages, inflammation, fibrosis

## Abstract

Diabetic kidney disease (DKD), a common microvascular complication of diabetes, has emerged as the leading cause of end-stage renal disease (ESRD), with its prevalence increasing annually, thereby constituting a global epidemiological crisis. Despite this, the search for effective therapeutic strategies remains challenging, underscoring the urgent need for innovative treatments. In recent years, numerous studies have highlighted the role of inflammation in DKD progression, with fibrosis serving as a critical mechanism driving DKD towards ESRD. Intervening in these core processes may reveal new avenues for the etiology and management of DKD. Macrophages, immune cells derived from monocytes, possess phagocytic and digestive functions and play integral roles in tissue and organ development, homeostasis, as well as tissue repair and regeneration. Emerging research suggests that macrophages are crucial in DKD progression and renal injury, thus attracting significant academic attention. This review highlights the role and mechanisms of macrophages in the inflammatory and fibrotic processes in DKD. It also explores current therapeutic approaches targeting macrophages. In light of recent research, we propose that macrophages-focused interventions present a promising therapeutic avenue for DKD.

## Introduction

As living standards improve and lifestyles change, the global prevalence of Diabetes Mellitus (DM) is rising rapidly, particularly in developing nations^1^, with an increasingly younger age of onset observed in China^2^. DM encompasses a group of metabolic disorders characterized by chronic hyperglycemia due to impaired insulin secretion or action^3^. Diabetic Kidney Disease (DKD) is among the most prevalent microvascular complications of diabetes, with an average annual incidence rate of 3%, typically manifesting 10-20 years post-DM onset^4^, ^5^. Without effective intervention and management, DKD can progress to chronic kidney disease (CKD). Presently, CKD affects 15-20% of adults globally^6^, including over 15% of adults in the United States^7^ and approximately 82 million adults in China^8^, substantially escalating the economic burden on governments and patients. As the primary cause of End-Stage Renal Disease (ESRD), early diagnosis and treatment of DKD are imperative^9^.

The pathogenesis of DKD is intricate, involving hemodynamic alterations, genetic predispositions, oxidative stress, and inflammatory responses. Inflammation is a pivotal factor in DKD progression, encompassing autoimmune disorders, complement dysregulation, and inflammatory activation, ultimately resulting in intrinsic renal cell damage, renal fibrosis, and diminished renal function^10^. Over the past two decades, extensive research has elucidated the mechanisms by which inflammation-induced diabetic kidney damage leads to renal fibrosis. Studies have demonstrated that macrophages are integral to the pathophysiological processes of DKD, particularly in inflammation and fibrosis^11^, ^12^.

**Figure 1:**
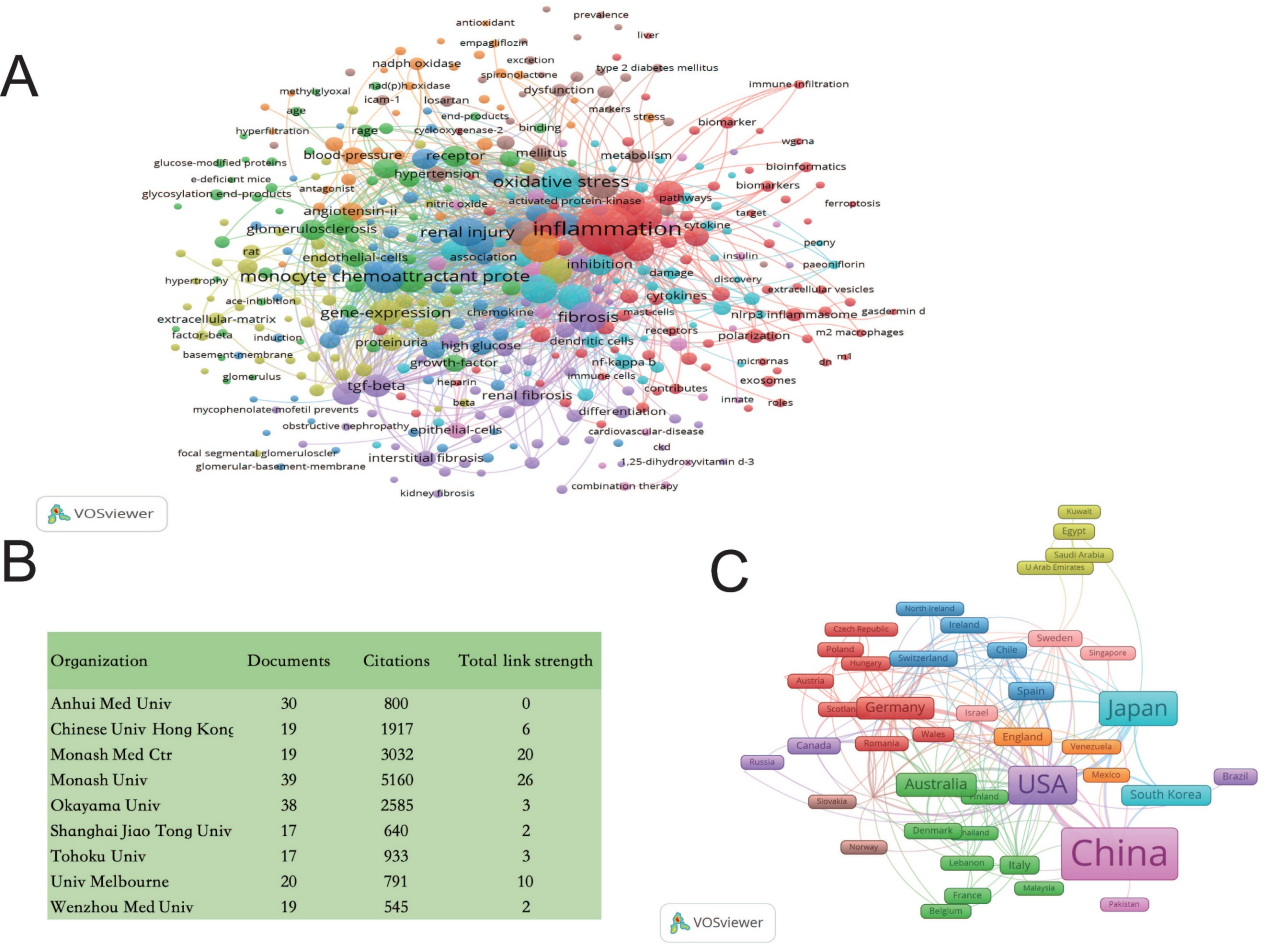
** Bibliometric Analyses of Macrophages in DKD.** Data were extracted from the Web of Science (www.isiknowledge.com/) database, and bibliometric analysis was performed using VOSviewer: A. Keyword Co-occurrence Network: This visualization includes 1,124 documents. The size of the round nodes represents the frequency of keyword appearances, indicating hotspots within the field. The connecting lines between nodes illustrate the association strength; thicker lines indicate more frequent co-occurrence in the same document. Node colors represent different clusters. B. Publishing Organizations: To provide a clearer understanding of the contributing organizations, the top 9 high-frequency organizations with a frequency greater than 17 were tabulated. C. Country Visualization: Countries with five or more publications are visualized. The width of the squares correlates with the number of publications, and thicker connecting lines indicate more frequent co-occurrence in the same publications. DKD: diabetic kidney disease.

Our team employed Web of Science (www.isiknowledge.com/) and VOSviewer software to analyze data, confirming the significant association between macrophages and inflammation and fibrosis in DKD. This research enriches our understanding and underscores the critical link between macrophages and DKD (Figure 1).

This paper reviews the role of macrophages in the inflammation and fibrosis of DKD, investigates potential therapeutic targets and strategies, and provides a theoretical basis for developing novel treatments aimed at mitigating the progression of DKD by targeting macrophages.

## The Pathophysiology and Pathogenesis of DKD

**Figure 2:**
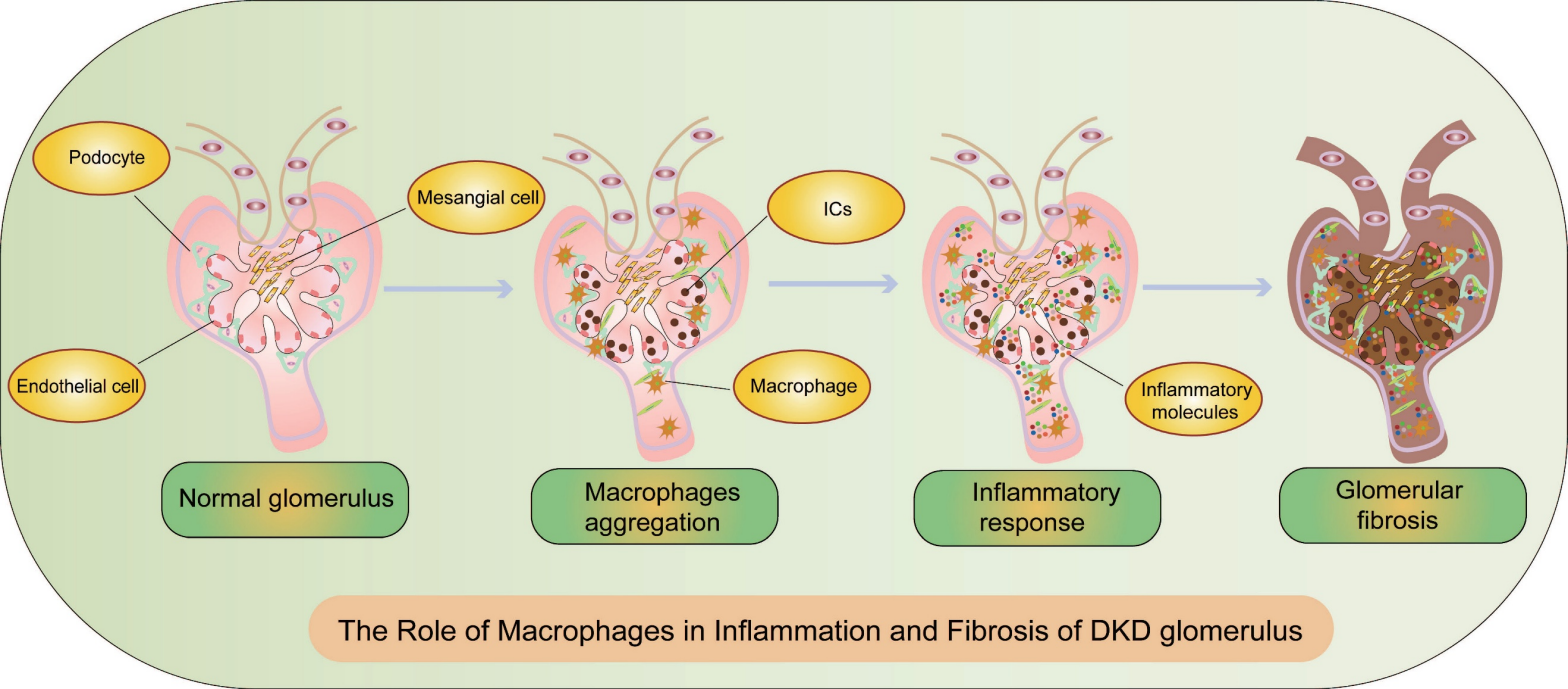
** The Role of Macrophages in Inflammation and Fibrosis of DKD Glomeruli.** During the progression of DKD, the activation of macrophages and sustained inflammation lead to fibrosis and CKD. CKD: chronic kidney disease, ICs: immune complex deposition.

According to the latest consensus from the American Diabetes Association (ADA) and the Kidney Disease: Improving Global Outcomes (KDIGO), DKD is defined by an estimated glomerular filtration rate (eGFR) <60 ml·min^-1^·(1.73 m^2^)^-1^ and/or an albumin-to-creatinine ratio (ACR)≥30 mg/g and/or other markers of kidney damage persisting for ≥3 months^13^. Pathological features of DKD, as outlined in the Chinese guidelines for diagnosis and treatment of diabetic kidney disease, include glomerular hypertrophy, mesangial matrix expansion, mesangial dissolution, Kimmelstiel-Wilson nodules, microaneurysms, exudative lesions, glomerular vascular proliferation, and tubulointerstitial lesions^14^. Inflammation and fibrosis are critical processes in DKD, characterized by renal basement membrane thickening and infiltration of inflammatory cells (Figure 2). The severity of tubular atrophy correlates with the extent of interstitial fibrosis, and the degree of interstitial fibrosis and inflammatory cell infiltration inversely correlates with renal survival, progressively deteriorating towards ESRD^15^ (Figure 2).

Although the pathogenesis of DKD is multifaceted and not entirely elucidated, it originates from hyperglycemia. Mechanisms underlying hyperglycemia-induced DKD include metabolic (hyperfiltration and hypertension), inflammatory, oxidative, genetic, and epigenetic factors^16^-^18^. Traditionally, DKD pathogenesis is attributed to aberrant glucose metabolism and increased intraglomerular pressure, resulting in hemodynamic abnormalities^19^. Hyperfiltration and glomerular hypertension can be precipitated by arteriolar vasodilation and activation of the renin-angiotensin-aldosterone system, leading to renal hemodynamic changes^20^. Oxidative stress results in elevated reactive oxygen species (ROS) formation in a high glucose (HG) environment, causing an imbalance in the oxidative/antioxidant system, cellular and tissue damage, extracellular matrix deposition, early microvascular injury, induction of cellular oxidative damage and apoptosis, and promotion of tubulointerstitial fibrosis^21^. Chronic inflammation and immune activation not only lead to renal damage and fibrosis but are also associated with genetic regulation of susceptibility to kidney injury in the pathogenesis of DKD^22^. Genetic and epigenetic modifications, such as DNA methylation, histone modifications, and miRNA regulation, also play a crucial role in the pathogenesis of DKD^23^. Despite the presence of multiple pathogenic mechanisms, failure to treat the condition promptly results in a singular outcome: renal fibrosis, ultimately leading to CKD.

## The Role of Renal Macrophages

Renal macrophages, located within the kidney, are crucial in preserving renal function, managing infections, and facilitating tissue repair^24^. It's well-established that macrophages originating from the embryo are primarily found in the renal medulla, whereas those from bone marrow are predominantly located in the cortex. Macrophages within the renal medulla perform a range of functions, including extending lengthy pseudopodia, crossing epithelial cell bodies, embedding within the tubular wall, and reaching into the lumen to clear granules, thereby preventing urinary blockage^25^. Comparisons of cells from diverse sources have shown that embryonically derived renal macrophages produce more inflammatory cytokines and have an enhanced phagocytic ability for immune complexes. For instance, Qin Xuebin and Liu Fengming's team from Dongguk University School of Medicine used a unique cell clearance mouse model and genetic lineage tracing technology to find that embryonically derived macrophages express higher levels of CD86 and tumor necrosis factor (TNF), indicating stronger immune response capabilities in acute kidney injury and nephritis models^26^.

It is well known that macrophages in different tissues and organs vary due to various reasons such as genetics and environment, which is known as tissue macrophages heterogeneity. Recently, there has been an increasing number of studies on the heterogeneity within tissue-specific macrophages populations. For instance, a recent article utilized single-cell transcriptomics to analyze mouse macrophages in five organs including the heart, liver, lung, kidney, and brain, and identified three common macrophage subpopulations: TLF (expressing TIMD4 and/or LYVE1 and/or FOLR2) macrophages maintained through self-renewal with minimal monocyte input; CCR2 (TIMD4++-LYVE1-FOLR2-) macrophages almost entirely replaced by monocytes, and MHC-IIhi macrophages (TIMD4-LYVE1-FOLR2-CCR2-), providing a common starting point for studying tissue macrophage heterogeneity^27^.

Serving as antigen-presenting cells (APCs), renal macrophages are key players in the immune response, demonstrating substantial phenotypic plasticity and adaptability to a variety of microenvironmental signals. Macrophages communicate bidirectionally with intrinsic renal cells. This communication leads to an increase in macrophage numbers, thereby enhancing their activation and causing potential damage to intrinsic renal cells through the secretion of inflammatory factors and the activation of ROS. On the other hand, intrinsic renal cells have specific immune properties and can produce various protein factors to attract macrophages, consequently promoting renal inflammation^19^, ^28^.

**Figure 3:**
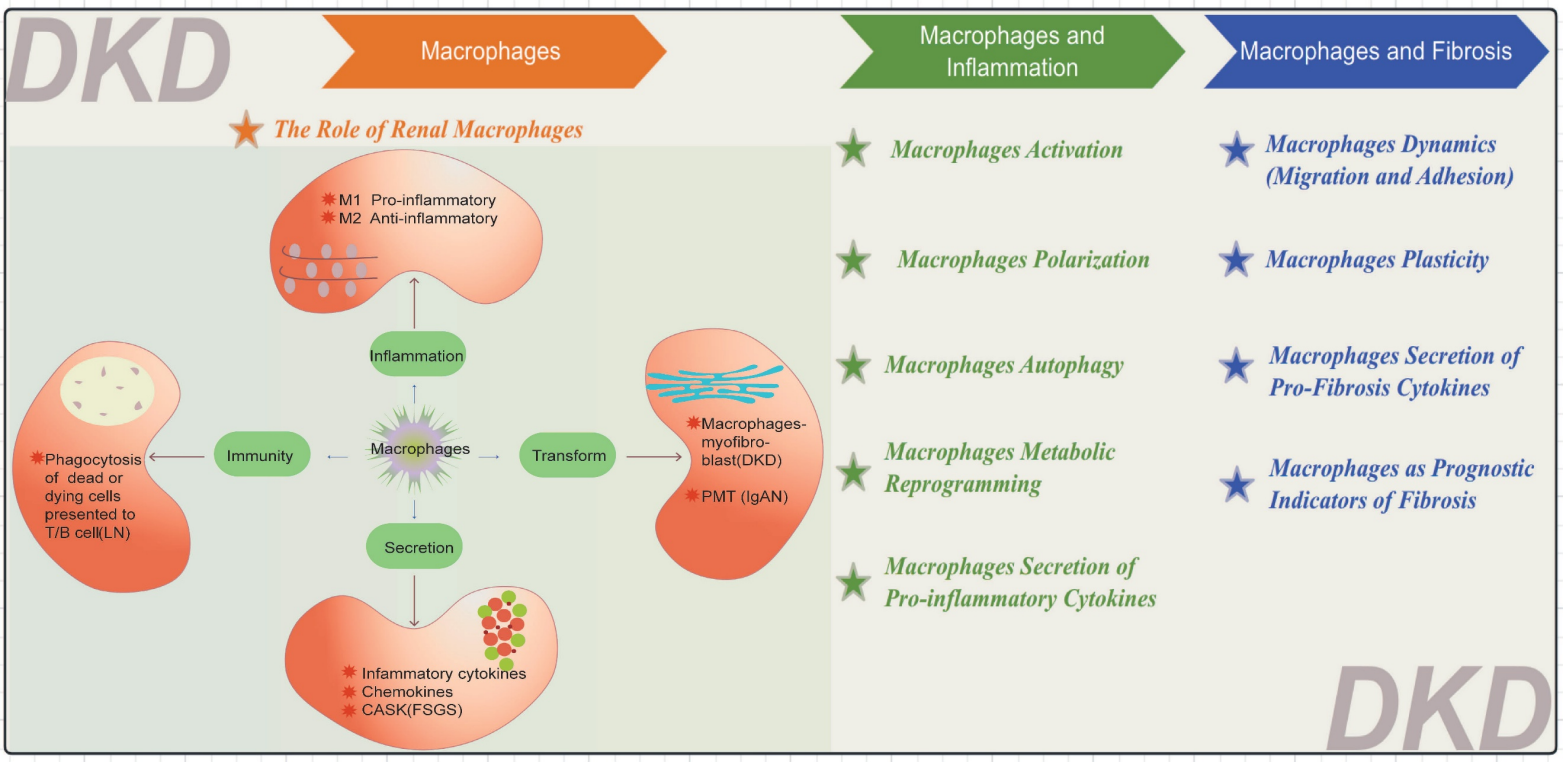
** Diverse Functions of Macrophages.** The functions of macrophages are categorized into four main areas: inflammation, secretion, immunity, and transformation. LN: lupus nephritis, IgAN: IgA nephropathy FSGS: focal segmental glomerulosclerosis, CASK: calcium/calmodulin-dependent serine/threonine kinase, PMT: podocyte-macrophage transdifferentiation.

Macrophages are pivotal in the pathogenesis of numerous renal diseases due to their participation in the inflammatory response (Figure 3). They primarily differentiate into M1 pro-inflammatory and M2 anti-inflammatory cells, as seen in DKD^29^. In DKD, cell damage and macrophages-myofibroblast transition (MMT) eventually lead to renal inflammation and fibrosis^30^. Macrophages can also secrete various inflammatory and fibrotic factors. In lupus nephritis (LN), macrophages phagocytose dead or dying cells^31^, ^32^, Subsequently, these cells present cellular remnants to T cells and B cells, fostering the production of autoantibodies by plasma cells and ultimately culminating in the deposition of immune complex deposition (ICs) within the kidneys^33^. In IgA nephropathy (IgAN), macrophages recognize and clear IgA immune complexes; however, excessive IgA deposition can overactivate macrophages, exacerbating renal inflammation. IgA1 can induce Nucleotide binding oligomerization domain-like pyrin domain-containing protein 3 (NLRP3) expression and initiate podocyte-macrophage transdifferentiation (PMT)^34^. Notably, within the past five years, a prospective study has elucidated the role of monocytes and M2 macrophages in secreting calcium/calmodulin-dependent serine/threonine kinase (CASK) via exosomes, impacting the integrity of the glomerular filtration barrier within focal segmental glomerulosclerosis (FSGS) kidneys^35^.

## The Role of Macrophages in DKD

Emerging research indicates that macrophages are the primary cells and crucial contributors to the progression of various kidney diseases, especially DKD^36^. To investigate the heterogeneity of renal macrophages in DKD, researchers conducted unsupervised clustering analysis on mononuclear phagocyte (MNP) cells from the kidneys of 3-month-old male diabetic OVE26 mice and non-diabetic littermate control mice. The analysis identified six distinct macrophage subpopulations: resident Mac, inflammatory Mac, IFNhi Mac, Mcr1hi Mac, Trem2hi Mac, and proliferating Mac, each exhibiting unique characteristics^37^. This heterogeneity and functional diversity among macrophages in DKD provide vital insights into their roles.

In the early stage of DKD, macrophages are recruited and differentiated by inflammatory factors after being stimulated by HG through various signaling pathways (such as phosphatidylinositol 3-kinase (PI3K) / protein kinase B (AKT) and Nuclear Factor kappa-light-chain-enhancer of activated B cells (NF-κB)), and secrete inflammatory and fibrotic factors, resulting in a series of reactions such as activation, polarization, migration and adhesion, transformation, and autophagy, causing fibrosis of intrinsic renal cells and ultimately leading to renal sclerosis and proteinuria^30^, ^38^.

### The Role of Macrophages in Inflammation of DKD

Inflammation is a central component of DKD's pathophysiology, orchestrating immune-mediated processes causing glomerular and tubulointerstitial damage, fibrosis, and progressive renal function decline^39^. The inflammatory processes include inflammatory cell recruitment, cytokine infiltration, formation of inflammatory bodies, and complement activation^40^ (Figure 2). Some research suggests that inflammation could become a promising target for DKD treatment by 2030^41^. Inflammatory macrophages are key players at every stage of DKD lesions. For instance, single-cell RNA sequencing revealed that Ras-related C3 botulinum toxin substrate 1(RAC1) plays a significant role in macrophage phagocytic activity in DKD, and impaired phagocytosis exacerbates inflammation^42^. Therefore, studying macrophages in the inflammatory process of DKD carries significant value.

#### Macrophage Activation

**Figure 4:**
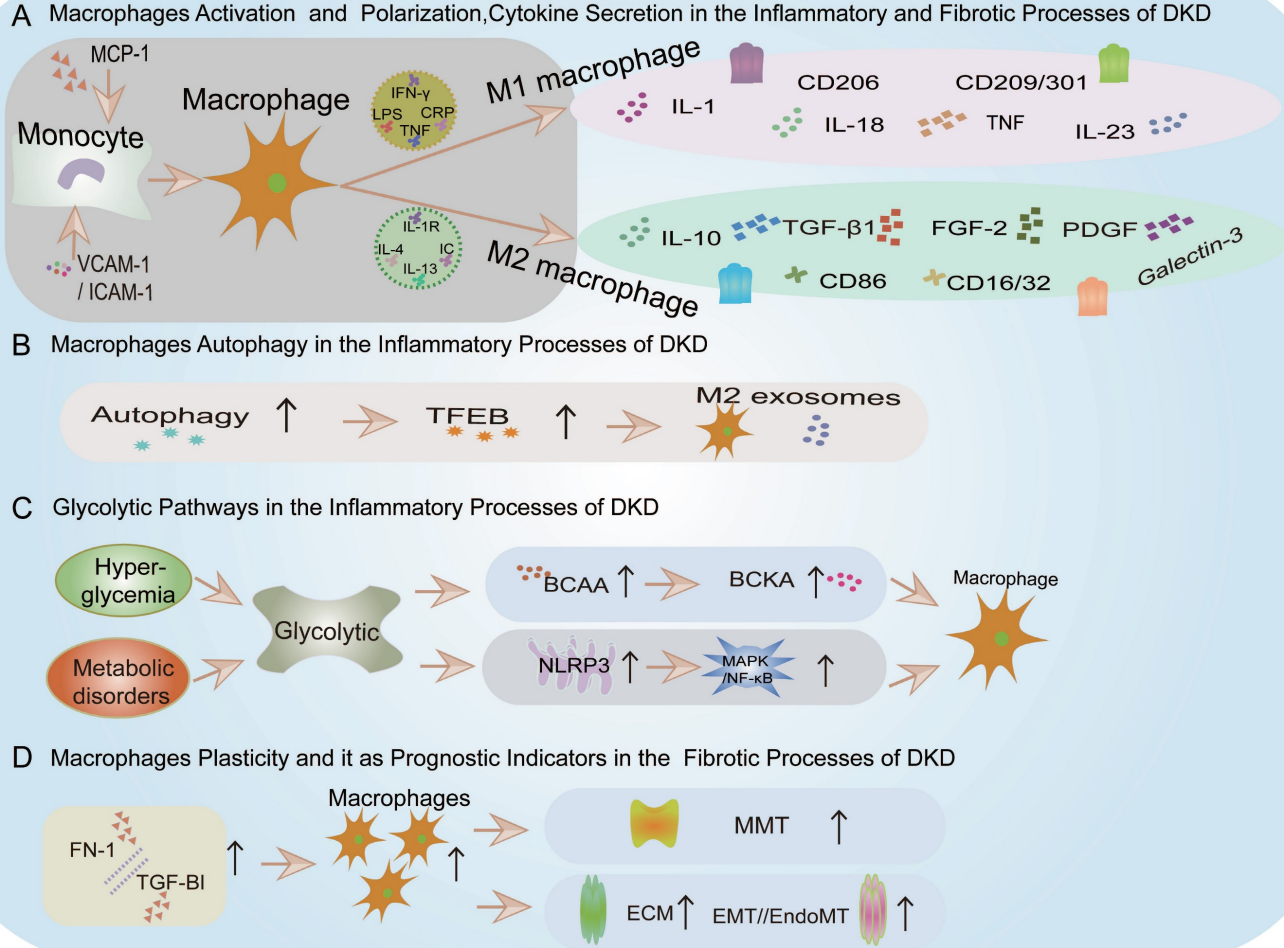
** Summary of Macrophages in Inflammation and Fibrosis of DKD.** A. Macrophage Activation and Polarization, Cytokine Secretion in the Inflammatory and Fibrotic processes of DKD. VCAM-1: Vascular Cell Adhesion Molecule-1, ICAM-1: Intercellular cell adhesion molecule-1, MCP-1: Monocyte chemoattractant protein-1, CCL2: Chemokine (C-C motif) ligand 2, IFN-γ: interferon-gamma, TNF: tumor necrosis factor, LPS: lipopolysaccharides, CRP:C-reactive protein, IL-1R: interleukin-1 receptor, FGF-2: fibroblast growth factor 2, PDGF: platelet-derived growth factor. B. Macrophage Autophagy in the Inflammatory processes of DKD. TFEB: The transcription factor EB. C. Glycolytic Pathways in the Inflammatory processes of DKD. BCAA: branched-chain amino acids, BCKA: branched-chain keto acids, NLRP3: Nucleotide binding oligomerization domain-like pyrin domain-containing protein 3, MAPK: Mitogen-activated protein kinase, NF-κB: Nuclear Factor kappa-light-chain-enhancer of activated B cells. D. Macrophage Plasticity and it as Prognostic Indicators in the Fibrotic processes of DKD. MMT: macrophage-myofibroblast transition, ECM: extracellular matrix, EMT: epithelial-mesenchymal transition, EndoMT: endothelial-mesenchymal transition, FN1: fibronectin 1, TGFBI: transforming growth factor beta induced.

Macrophages can be polarized into either the M1 or M2 phenotypes (Figure 4). In the context of DKD, classically activated M1 macrophages display pro-inflammatory characteristics essential for host defense and tissue injury^43^. These M1 macrophages are typically induced by stimuli such as interferon-gamma (IFN-γ), lipopolysaccharides (LPS), C-reactive protein (CRP), or cytokines like TNF^30^ (Figure 4). Common markers for M1 macrophages include CD86, CD16/32, major histocompatibility complex II (MHC II), interleukin-1 receptor (IL-1R), human leukocyte antigen-DR (HLA-DR), and CD197(Figure 4). Conversely, alternatively activated M2 macrophages in DKD exhibit anti-inflammatory properties, facilitating tissue repair and inflammation resolution^30^, ^44^. M2 macrophages are further subdivided into M2a (induced by IL-4 or IL-13), M2b (induced by immune complexes and Toll-like receptors or IL-1R ligands), and M2c (induced by IL-10 and glucocorticoids) (Figure 4). M2a macrophages are robust anti-inflammatory and tissue repair agents, whereas M2b and M2c macrophages, which secrete IL-10, help regulate inflammation without synthesizing extracellular matrix, functioning as regulatory macrophages. Common markers for M2 macrophages include CD206, CD209, and CD301(Figure 4).

Genetic modification of transcription factors and phosphorylated glycoproteins can also affect macrophage activation. In one study, the authors considered that the transcription factor nuclear factor-erythroid 2 related factor 2 (Nrf2) might affect macrophage activation and thereby influence DKD by constructing db/db *Nrf2* knockout (KO) mice and detecting the expression of the macrophage's marker CD36 in diabetic patients^45^. In DKD, high blood sugar can stimulate the upregulation of myeloid-related protein 8 (MRP8) activate macrophages, MRP8 mediated by fetuin A and the transcription factors activator protein 1 (AP-1) and CCAAT enhancer binding protein beta (CEBP/β), and change the expression of exosomal miRNAs in macrophages (including miR-193a-3p, miR-1260b, and miR-3175) through the NF-κB/ Janus kinase-signal transducer and activators of transcription (JAK-STAT) pathway^30^. Among all human tissues, the kidney has the highest content of osteopontin (OPN, a secreted pleiotropic phosphorylated glycoprotein). Overexpression of OPN may lead to the activation and recruitment of macrophages, resulting in significant accumulation of macrophages and potentially affecting the inflammatory and fibrotic processes of DKD through the expression of transforming growth factor-β (TGF-β), extracellular signal-regulated protein kinases (ERK)/mitogen-activated protein kinase (MAPK), and Jun N-terminal kinase (JNK)/MAPK signaling pathways, causing renal damage^46^.

This binary classification is not fixed; macrophages can swiftly alter their phenotypes in response to local microenvironmental cues. Modulating macrophage activity to alleviate renal inflammation and fibrosis presents a promising therapeutic strategy^47^.

#### Macrophage Polarization

Macrophage polarization involves the differentiation of mature macrophages into distinct functional phenotypes in response to specific microenvironmental signals. Dysregulation in macrophage polarization and phenotypic transitions plays a significant role in the pathogenesis and progression of DKD. Chronic hyperglycemia and associated metabolic disorders bias macrophages towards the M1 phenotype, exacerbating tissue damage^29^. For instance, Fu *et al.* utilized single-cell transcriptomic analysis of monocytes and CD45-rich kidney immune cells in type 1 diabetic OVE26 mice, observing an increased prevalence of the M1-like inflammatory phenotype over time^37^.

Although the precise mechanisms by which M2 macrophages mitigate DKD progression and facilitate kidney repair are not fully elucidated, certain therapeutic agents have been shown to modulate serum cytokine and acute-phase protein levels. These agents can induce a shift from pro-inflammatory M1 macrophages to anti-inflammatory M2 macrophages, thereby dampening the inflammatory response^43^. A study analyzing research from January 1998 to May 2019 on diabetic animals with kidney endpoints highlighted cell regeneration therapies, such as exosome release and cell-to-cell interactions by mesenchymal stromal cells (MSCs), as mechanisms to reactivate the endogenous repair system. MSCs release soluble mediators with anti-fibrotic, anti-apoptotic, pro-angiogenic, and anti-inflammatory properties that promote kidney repair. Systematic evaluations and meta-analyses have demonstrated that macrophages phagocytizing apoptotic MSCs promote an anti-inflammatory phenotype switch associated with IL-10 release^48^. Increasing attention is being paid to the imbalance between M1 and M2 macrophage populations in the diabetic kidney, with pro-inflammatory M1 macrophages predominating and driving kidney inflammation and fibrosis^49^. Macrophage polarization is critical for pathogen defense, inflammation regulation, tissue repair, and homeostasis maintenance.

#### Macrophage Autophagy

Autophagy is a cellular mechanism where double-membrane vesicles encapsulate cytoplasmic components and organelles, forming autophagosomes that fuse with lysosomes to create autolysosomes, thus degrading their contents to meet cellular metabolic demands and renew organelles. In DKD, autophagy serves as a crucial regulatory factor, primarily by attenuating kidney inflammation. The transcription factor EB (TFEB) plays a key role in autophagy regulation, influencing macrophage activation and polarization (Figure 4).

Studies indicate that autophagy regulates macrophage phenotype transitions, while its inhibition enhances macrophage adhesion and migration^50^. For instance, some researchers used male BALB/c mice (6-8 weeks old) and induced diabetes by intraperitoneal injection of streptozotocin (STZ) (150 mg/kg), using a blood glucose level of ≥16.7 mmol/L as the diagnostic standard. Starting from the fourth week after diabetes onset, they administered MSCs via tail vein injection (5 × 10^5^ cells in 0.1 mL of physiological saline, once every two weeks, for a total of three injections) and euthanized the mice 12 weeks post-diabetes onset. To analyze infiltrating macrophages (Mφ) in the kidneys, they minced the kidney tissue and separated the single-cell suspension using collagen IV (1 mg/mL) and Deoxyribonuclease (DNase 0.1 mg/mL) to form a turbid mixture. Flow cytometry was used to analyze the phenotype using monoclonal antibodies: F4/80-APC, CD206-PE, and CD11c-FITC. The results showed that MSCs induce an M2 phenotype in Mφ by enhancing TFEB-mediated autophagy, thereby alleviating DKD^51^. Additionally, some researchers used high glucose conditions to induce a podocyte injury model and found that M2 macrophages-derived exosomes can also activate macrophage autophagy, improving DKD-induced podocyte injury^52^ (Figure 4).

#### Macrophage Metabolic Reprogramming

Macrophage responses can be stimulated through appropriate bioenergetic metabolic pathways. Macrophage metabolic reprogramming refers to the regulation of metabolic pathways to influence the responses of macrophages^53^, ^54^. In DKD, it specifically refers to macrophage's glycolytic pathways.

Embryonically derived kidney macrophages exhibit an enhanced glycolytic capacity and glucose uptake proficiency, allowing them to efficiently harness energy through glycolysis under pathological circumstances. However, aberrant glycolysis can precipitate renal damage. For instance, researchers employed a db/db mouse model, administering renal tubular epithelial cell (HK-2) extracellular vesicles (EVs) via the tail vein, followed by treatments with human serum albumin and the glycolysis inhibitor 2-DG. They subsequently assessed the expression of glycolytic enzymes in macrophages utilizing Reverse Transcription-quantitative Polymerase Chain Reaction (RT-qPCR), western blotting, and immunohistochemistry. The findings indicated that EVs from HK-2 promote macrophage glycolysis by stabilizing Hypoxia-inducible factor-1α (HIF-1α), thereby activating genes related to inflammation and fibrosis, ultimately resulting in albuminuria; albuminuria, in turn, induces renal macrophage glycolysis^55^. And, some scholars have used cell cycle checkpoint kinase 2 (Chk2)-deficient mice models to study and found that ROS, as a signaling molecule, can promote glycolysis and M1 polarization of macrophages through Chk2^56^.

Chronic hyperglycemia and metabolic disorders can engender glycolysis anomalies in DKD, fostering a pro-inflammatory renal milieu marked by macrophage activation and pro-inflammatory cytokine secretion^29^ (Figure 4). These glycolytic disturbances also activate the NLRP3 inflammasome within various intrinsic renal cells (mesangial cells, podocytes, tubular epithelial cells, and infiltrating macrophages), a critical multiprotein complex in DKD that modulates innate immune responses and inflammatory cascades^57^, ^58^ (Figure 4).

Additionally, glycolytic metabolites may excessively activate macrophages, causing renal injury. For example, studies in db/db mouse models revealed that dysregulation of branched-chain amino acids (BCAA) metabolism could lead to the accumulation of branched-chain keto acids (BCKA), triggering excessive oxidative stress in macrophages, exacerbating inflammation, and inflicting renal tissue damage^59^(Figure 4).

#### Macrophage Secretion of Pro-Inflammatory Cytokines

M1 macrophages secrete pro-inflammatory mediators that drive leukocyte recruitment, endothelial dysfunction, extracellular matrix remodeling, and renal fibrosis, worsening renal dysfunction and proteinuria in DKD patients^44^. These M1 macrophages exhibit notable pro-inflammatory activity, predominantly producing IL-1, IL-18, IL-23, IFN-γ, TNF-α, ROS, nitric oxide (NO), and other pro-inflammatory cytokines (Figure 4). While these cytokines combat pathogen invasion, they also inflict tissue damage. For instance, studies using macrophage inflammatory protein-1β (MIP-1β) KO mice in comparison to WT mice demonstrated that inhibition or loss of MIP-1β in DKD protected podocytes, regulated renal inflammation, and mitigated glomerulosclerosis and fibrosis^60^. Moreover, the pro-inflammatory cytokine milieu in diabetic kidneys, characterized by elevated IL-1β, IL-6, and TNF-α levels, further amplifies the activation and polarization of M1 macrophages.

Conversely, the presence of M2 macrophages in diabetic kidneys is linked with tissue repair, resolution of inflammation, and regulation of fibrosis^43^. For example, studies using C57BL/6J WT mice and Ras guanine nucleotide-releasing protein-4 (RasGRP4) KO mice subjected to a high-fat diet combined with streptozotocin (60 mg/kg) to induce diabetes showed that RasGRP4 KO mice exhibited reduced macrophage infiltration, lower expression of inflammatory mediators such as IL-6, TNF-α, intercellular cell adhesion molecule-1 (ICAM-1), and vascular cell adhesion molecule-1 (VCAM-1), as well as decreased NLRP3 inflammasome and MAPK/ NF-κB signaling pathways. Additionally, the adhesion function of peripheral blood mononuclear cells (PBMCs) was diminished in RasGRP4 KO mice^61^ (Figure 4). However, the reparative function of M2 macrophages may be compromised in a diabetic environment, leading to unresolved inflammation and progressive renal damage.

Many signal transduction pathways are also crucial in macrophages-mediated inflammation and fibrosis in DKD. For instance, HG activates the reactive ROS-p38 MAPK pathway in macrophages to release TNF-α, thereby causing kidney damage in DKD^30^.The PI3K/Akt signaling pathway is associated with M1 polarization in macrophages isolated from DKD patients in the NF-κB signaling cascade, and the PI3K/Akt pathway is regulated by the long non-coding RNA (lncRNA) LINC00323^38^.The PI3K/Akt signaling pathway is involved in the inhibition of peroxisome proliferator-activated receptor γ (PPARγ) in the neutrophil gelatinase-associated lipocalin (NGAL) signaling cascade, which is related to the polarization of the M2 anti-inflammatory phenotype and increases the production of anti-inflammatory IL-10 by macrophages^38^, ^62^.

### The Role of Macrophages in Fibrosis of DKD

In DKD, sustained inflammation incites cell activation, endothelial-mesenchymal transition, and cellular infiltration, culminating in fibrosis and CKD^40^(Figure 2). During the progression of DKD, macrophages undergo a series of pathological transformations, leading to irreversible fibrotic changes in the glomeruli and renal interstitium. Deciphering the intricate pathways leading to glomerular fibrosis may unveil novel strategies for preventing or mitigating renal disease. The involvement of M2 macrophages in renal tissue fibrosis remains a topic of debate.

#### Macrophage Dynamics (Migration and Adhesion)

Macrophage recruitment is primarily elicited by initial inflammatory signals, prompting their proliferation and differentiation into distinct functional phenotypes^49^. Prior to recruitment, there is an upregulation of VCAM-1, ICAM-1, and Monocyte chemoattractant protein-1 (MCP-1)/Chemokine (C-C motif) ligand 2 (CCL2), which facilitate monocyte (macrophages) migration (Figure 4). For instance, research has shown that podocytes, when stimulated with growth hormone and analyzed via RNA sequencing, can secrete TNF-α, prompting monocyte differentiation into macrophages and enhancing macrophage migration, ultimately resulting in inflammation and fibrosis of the kidneys associated with DKD^63^.

In DKD, a prolonged hyperglycemic environment fosters macrophage accumulation and infiltration, induces cytokine/chemokine release, activates monocyte/macrophage recruitment, and accelerates various pathological processes within the kidneys^30^. For example, studies using two DKD animal models demonstrated that elevated glucose levels led to the upregulation of Cullin4B (CUL4B) by inhibiting the expression of miR-194-5p, thereby increasing integrin α9 (ITGA9) levels, promoting macrophage migration and adhesion^64^.

#### Macrophage Plasticity

Macrophages secrete pro-inflammatory cytokines, aggravating tissue damage and exacerbating inflammation, promoting myofibroblast proliferation, and recruiting fibroblasts^44^. At the injury onset, M1 macrophages are the predominant cell type involved. In a simulated diabetic kidney microenvironment, M1 macrophages participate in repair and remodeling, differentiate into fibroblasts, and mitigate kidney damage^65^. These activated M1 macrophages degrade extracellular matrix (ECM) metalloproteinases, inhibit epithelial-mesenchymal transition (EMT) or endothelial-mesenchymal transition (EndoMT), causing cell damage, ultimately leading to kidney fibrosis (Figure 4). For example, single-cell RNA sequencing of 27,424 kidney cells revealed TGF-β1+Arg1+ fibrosis-related macrophages in DKD^66^.

M2 macrophages are capable of activating myofibroblasts through multiple mechanisms. Beyond indirectly fostering fibrosis by recruiting, proliferating, and activating fibroblasts, macrophages can directly induce fibrosis via a process known as MMT^47^. MMT is a process where macrophages recruited from the bone marrow can be directly transformed into myofibroblasts in injured kidneys through a Src-centered regulatory network driven by TGF-β1-Smad3 signaling^67^.

The combination of cell injury and MMT ultimately precipitates diabetic kidney injury, renal inflammation, and fibrosis^30^.

#### Macrophage Secretion of Pro-Fibrosis Cytokines

M2 macrophages secrete factors such as TGF-β1, fibroblast growth factor 2 (FGF-2), platelet-derived growth factor (PDGF), and galectin-3, which facilitate the proliferation, survival, and activation of myofibroblasts, enhance tissue repair, and promote angiogenesis and extracellular matrix remodeling^44^(Figure 4). Current research in this realm remains limited, indicating a potential avenue for future investigations.

#### Macrophages as Prognostic Indicators of Fibrosis

During the fibrotic stage of the kidney, macrophages predominantly exhibit an M2 phenotype. The accumulation of these macrophages in the renal interstitium serves as a critical prognostic marker, reflecting the severity of renal impairment and the extent of interstitial fibrosis^68^.

For example, a study by Huang, B., W. Wen, and S. Ye involved 178 type 1 diabetes patients with microalbuminuria complications, each hospitalized at least twice over a period of more than 24 months at the Department of Endocrinology, First Affiliated Hospital of the University of Science and Technology of China. The patients were categorized based on urinary albumin-creatinine ratio (UACR) stages and 25-hydroxyvitamin D levels: G1 (N=45), <10 ng/mL; G2 (N=80), 10-20 ng/mL; and G3 (N=53), ≥20 ng/mL. Using the CIBERSORT algorithm to assess immune cell infiltration in advanced renal tissue, they discovered that reduced M2 macrophage infiltration could predict a heightened risk of DKD proteinuria progression and a decline in eGFR^69^. An additional analysis of the GSE96804 dataset using the CIBERSORT algorithm revealed a significant increase in macrophages in DKD renal tissue samples. Fibronectin 1 (FN1) and transforming growth factor beta-induced (TGFBI) exhibited strong positive correlations with M2 macrophages, indicating their roles in macrophage-induced immune response and fibrosis. These findings suggest that FN1 and TGFBI could serve as promising biomarkers for the diagnosis and treatment of DKD patients^70^(Figure 4).

## 5. Current Methods of Macrophage-Targeted Therapy

**Figure 5:**
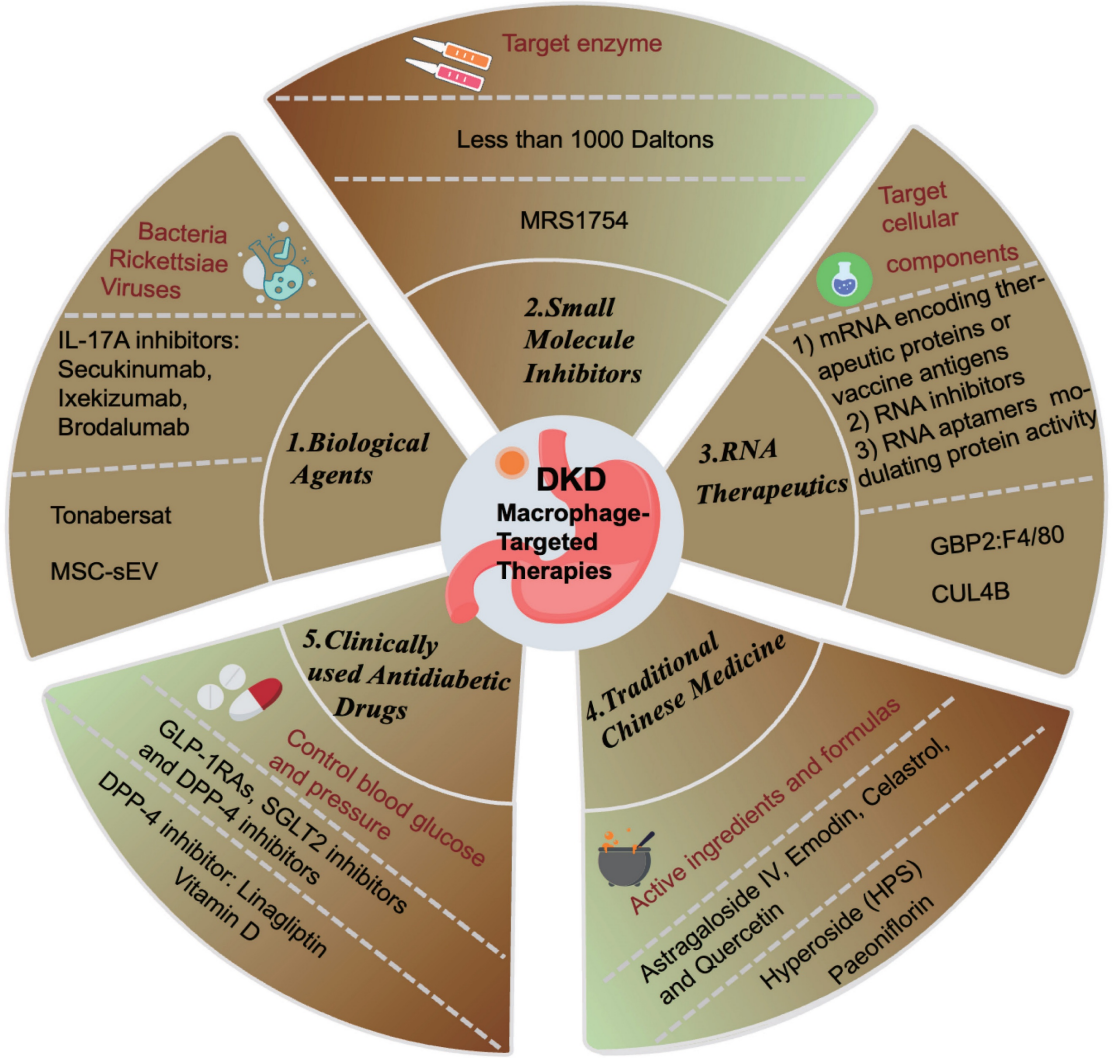
** Summary of Macrophage-Targeted Therapies.** Biological Agents: MSC-sEV: mesenchymal stem cell-derived extracellular vesicles; Small molecule inhibitors; RNA therapeutics: CUL4B: Cullin4B; Traditional Chinese medicine; Clinically used antidiabetic drugs; GLP-1RAs: glucagon-like peptide-1 receptor agonists, SGLT2: sodium-glucose cotransporter-2, DPP-4: dipeptidyl peptidase-4.

According to the KDIGO guidelines, current treatment strategies for DKD primarily focus on controlling blood glucose and blood pressure, along with other symptomatic treatments^71^. Commonly prescribed medications include glucose-lowering agents such as glucagon-like peptide-1 receptor agonists (GLP-1RAs), sodium-glucose cotransporter-2 inhibitors (SGLT2i), and dipeptidyl peptidase-4 (DPP-4) inhibitors. Blood pressure management drugs commonly used are renin-angiotensin-aldosterone system (RAAS) blockers, angiotensin-converting enzyme inhibitors (ACEi), and endothelin receptor antagonists^72^ (Figure 5). The local RAS promotes monocyte adhesion to renal endothelial cells and increases the production of MCP-1 and OPN in renal tubular cells, thereby facilitating monocyte entry into the kidneys. Within the kidneys, pro-inflammatory cytokines and RAS induce the differentiation of macrophages into the M1 pro-inflammatory phenotype, which predominantly contributes to renal lesions in DKD^73^. Research suggests that these drugs impact macrophages either directly or indirectly, and can be categorized accordingly.

### Biological Agents

Immunobiological agents encompass biological products derived from microorganisms (bacteria, rickettsiae, viruses, etc.) and their metabolic products with effective antigen components, animal toxins, or human/animal blood or tissues, used for the prevention, treatment, and diagnosis of corresponding infectious or other related diseases, such as vaccines and antibodies (Figure 5). For instance, targeting IL-17A for DKD therapy is contentious. Several animal studies have demonstrated that IL-17A KO alleviated renal inflammation and reduced interstitial fibrosis in DKD mice^74^-^76^, and intraperitoneal injection of IL-17A monoclonal antibody reduced proteinuria and pathological fibrosis^75^. However, recent studies suggest that IL-17A may mitigate renal inflammation and fibrosis by regulating autophagy or macrophage phenotype^77^. A 2021 study revealed that IL-17 deficiency exacerbated STZ-induced diabetic nephropathy mice by impairing autophagic responses^78^. Despite ongoing debates regarding IL-17A inhibitors, drugs such as secukinumab, ixekizumab, and brodalumab have been developed, but they have not yet advanced to clinical trials for DKD patients (Figure 5).

Additionally, researchers employed human transcriptome data from the Nephroseq (www.nephroseq.org, University of Michigan, Ann Arbor, MI) repository and cultured primary human renal proximal tubule epithelial cells (RPTECs) and monocyte-derived macrophages (MDMs) as a DKD model. Findings indicated that Tonabersat, a Cx43 hemichannel blocker, inhibited glucose/cytokine-induced ATP release mediated by aberrant connexin 43 (Cx43) hemichannels (Figure 5). This inhibition led to reduced inflammatory marker expression, decreased NLRP3 inflammasome activation in RPTECs, diminished macrophage inflammatory marker expression (IL-1β, MCP-1, and TNFα), and reduced macrophage migration^79^.

Recent studies have shown that mesenchymal stem cell-derived extracellular vesicles (MSC-sEV) can act as nanotherapeutic agents targeting renal tissue. MSC-sEV loaded with casein kinase 1 delta (CK1δ) and β-transducin repeat-containing protein (β-TRCP) can alleviate renal fibrosis by promoting Yes-Associated Protein (YAP) ubiquitination and degradation, thereby inhibiting YAP activity and ameliorating renal fibrosis. This presents a novel and effective therapeutic approach for anti-fibrotic treatment^66^.

### Small Molecule Inhibitors

Small molecule inhibitors typically target enzymes by binding directly to them, reducing their activity, or hindering biochemical reactions by competing with substrates, altering protein structures, or inhibiting protein conformational changes. These inhibitors generally have a molecular weight of less than 1000 Daltons, exhibit high selectivity and cellular permeability, and are widely utilized in life sciences and clinical research (Figure 5). Most clinically used drugs are predominantly composed of small molecule inhibitors. Researchers isolated glomeruli from DKD rats under non-diabetic conditions, DKD, and treatment with MRS1754, a selective A2B adenosine receptor antagonist, to perform RNA-seq studies and ELISA assays. The results indicated that MRS1754 downregulated the expression and secretion of chemokines CCL2, CCL3, CCL6, and CCL21 in DKD rat glomeruli, reduced macrophage infiltration, and promoted M2 macrophage polarization. Neutralizing antibodies against CCL2, CCL3, and CCL21 significantly inhibited macrophage migration induced by DKD glomerular conditioned medium, suggesting the anti-fibrotic potential of MRS1754^80^(Figure 5).

And, CCX140-B is a small molecule CCR2 antagonist that can inhibit CCR2 and block MCP-1-dependent monocyte and macrophage migration and activation. In a study that recruited 332 patients with type 2 diabetes aged 18-75 years with proteinuria from 78 research centers in Belgium, the Czech Republic, Germany, Hungary, Poland, and the UK, all patients were divided into three groups: receive placebo, 5 mg CCX140-B, and 10 mg CCX140-B, and were given the drug for 52 weeks. The data indicated that CCX140-B has a renal protective effect on patients with type 2 diabetic kidney disease^81^.

### RNA Therapeutics

RNA therapeutics constitute a distinct class of drugs, separate from small molecules and antibodies. These therapeutics can target cellular components such as mRNA and ncRNA to silence genes and reduce the expression of target proteins, thereby offering potential treatments for various diseases (Figure 5). Additionally, RNA-based drugs facilitate the development of novel vaccines and protein replacement therapies. Currently, three primary categories of RNA therapies are recognized: 1) mRNA encoding therapeutic proteins or vaccine antigens, 2) RNA inhibitors like siRNA, miRNA, and ASO targeting pathogenic RNA, and 3) RNA aptamers modulating protein activity (Figure 5).

Researchers analyzed gene microarray datasets from the Gene Expression Omnibus (GEO) to identify 16 key genes with high modular connectivity related to M1 macrophages. By integrating CIBERSORT, Weighted Gene Co-expression Network Analysis (WGCNA), and DEGs, they identified hub genes CASP1, MS4A4A, CD53, and GBP2 using LASSO regression analysis. Subsequent experiments in HG cultured THP-1 cells showed that siRNA targeting GBP2 colocalizes with the macrophage's marker F4/80, revealing that GBP2 promotes M1 macrophage polarization via activation of the Notch1 signaling pathway in DKD^82^(Figure 5). DKD treatment strategies include stimulating M2 macrophages while inhibiting M1 macrophages. For instance, studies in two DKD mouse models demonstrated that myeloid-specific CUL4B deficiency increases M2 macrophage numbers, thereby mitigating diabetes-induced kidney damage and fibrosis^64^(Figure 5).

In a 2021 article, 90 clinical samples from DKD patients diagnosed and treated between January and September 2020 were analyzed. The study revealed an increase in the expression of inflammatory factors TNF-α and IL-6. PCR and Western blot analyses confirmed elevated levels of the M1 marker protein CD86. Bioinformatics identified a key target, Long Intergenic Non-Protein Coding RNA 323 (LINC00323), which was also found to be highly expressed in patient blood samples. Further analysis at the cellular and molecular biology levels, as well as in animal models, indicated that LINC00323 mediates M1 macrophage polarization through the PI3K/AKT signaling pathway^83^.

RNA-targeted therapeutics are poised to become a pivotal area in biopharmaceutical innovation, despite the inherent challenges of RNA degradation and short half-life *in vivo*.

### Traditional Chinese Medicine

The rapid advancement of traditional Chinese medicine (TCM) in China has shown promise in mitigating adverse reactions associated with Western medicines and enhancing therapeutic efficacy in diabetes management. TCM demonstrates superior symptom improvement, stable efficacy, fewer side effects, and reduced costs. Research on TCM for DKD has evolved from isolated case studies to large-scale, systematic clinical trials, affirming the regulatory impact of compound formulas, single herbs, and monomers on key pathological mechanisms such as kidney inflammation, apoptosis, and fibrosis (Figure 5). Core active substances like astragaloside IV, emodin, celastrol, and quercetin have been identified, significantly contributing to innovative DKD drug development, reducing urinary protein levels, and delaying disease progression.

TCM active ingredients and formulas can modulate DKD progression by influencing macrophage polarization^84^ (Figure 5). For example, Hyperoside (HPS) has been shown to induce macrophage transition from the M1 to M2 phenotype, thereby inhibiting pro-inflammatory macrophage infiltration in the kidneys and increasing anti-inflammatory cytokine surface molecule expression (Figure 5). This modulation improves albuminuria and mesangial matrix expansion in DKD mice, alongside notable improvements in fasting blood glucose levels, hyperlipidemia, and body weight^85^. Additionally, paeoniflorin has demonstrated efficacy in reducing macrophage infiltration and inflammatory cytokine expression, enhancing kidney function, and ameliorating histological damage in diabetic mouse kidneys^86^.

### Clinically Used Anti-Diabetic Drugs

Clinically used anti-diabetic drugs such as GLP-1RAs, SGLT2 inhibitors, and DPP-4 inhibitors are effective in lowering blood glucose levels, with unique benefits in reducing urinary protein and protecting kidney function, making them particularly suitable for diabetic nephropathy patients (Figure 5).

For instance, the DPP-4 inhibitor linagliptin can degrade various chemokines and peptide hormones involved in immune regulation (Figure 5). DPP-4 is implicated in macrophage-mediated inflammation and insulin resistance. Utilizing fluorescence-activated cell sorting (FACS), we quantified DPP-4 (+) macrophages in lean and obese mice, as well as in the white adipose tissue (WAT) of diet-induced obese (DIO) mice. The findings revealed that DPP-4 can modulate macrophage polarization (M1/M2), thereby mitigating obesity-related insulin resistance and inflammation, and consequently reducing the damage from diabetic nephropathy^87^.

In a separate study, Sprague-Dawley (SD) rats were allocated into four groups: (a) normal control (NC), (b) 1,25-dihydroxyvitamin D3 treatment group, (c) DKD group, and (d) DKD with 1,25-dihydroxyvitamin D3 treatment group. Diabetes was induced via a single intraperitoneal injection of STZ at a dose of 58 mg/kg in 0.1M citric acid buffer (pH 4.5). Post-diabetes induction, 1,25-dihydroxyvitamin D3 was administered orally at 0.1 µg/kg daily for 18 weeks. Additionally, RAW264.7 macrophages were incubated with 30 mM glucose for 48 hours, with or without 1,25-dihydroxyvitamin D3 at a concentration of 10^-8^ mol/L. Analysis of rat kidney tissue and macrophages indicated that 1,25-dihydroxyvitamin D3 inhibits the shift of macrophages towards the M1 phenotype via the STAT-1/ Triggering Receptor Expressed on Myeloid Cells 1 (TREM-1) pathway^88^(Figure 5).

These studies underscore that targeting macrophages offers a promising strategy for DKD treatment, emphasizing the pivotal role of macrophages. However, such therapies necessitate vigilant monitoring for potential side effects, including immunosuppression. Despite encouraging results in animal models, most clinical trials in DKD patients have not demonstrated significant improvement, likely due to differences between human and murine immune systems. In patients with DKD, macrophages exhibit significant heterogeneity, encompassing multiple subtypes whose phenotypes and functions can vary substantially depending on the tissue microenvironment. Preclinical studies often target only one or a few macrophage subtypes, potentially overlooking the effects on other subtypes. The metabolic processes of drugs within the body are complex, posing a significant challenge in precisely delivering targeted therapies to macrophages at the lesion site in the kidneys. In clinical practice, physiological barriers such as the kidney's filtration function and the vascular endothelial barrier can impede the effective delivery of drugs to target macrophages.

How can we bridge the gap between preclinical research and clinical practice? First, we should develop animal models that more closely mimic the pathological characteristics of human DKD. For instance, constructing gene-edited mouse models with specific genetic mutations relevant to human kidneys can help study the effects of drugs on macrophages within different genetic backgrounds. With advancements in multi-omics technologies, a comprehensive analysis of macrophage heterogeneity in the kidneys of DKD patients can be conducted. Currently, omics technologies mainly include phosphoproteomics, single-nucleus RNA sequencing, or metabolomics^89^-^91^.

Precision medicine, which integrates modern technological approaches with traditional medical methods, aims to maximize specific and personalized treatments for patients. For example, researchers have analyzed transcriptome data from renal tissues of DKD patients (Gene Expression Omnibus) and proteomic analyses of urine from diabetic patients, discovering an elevated abundance of peroxiredoxin 2 (PRDX2) in DKD. Further experimental studies revealed that PRDX2 promotes M1 polarization of macrophages and enhances their migration and phagocytic abilities through toll-like receptor 4 (TLR4), providing a basis for further research on targeting macrophages for the diagnosis and treatment of DKD^92^.

Researchers utilized metabolomics methods to analyze the plasma and urine of 8-week-old WT and meprin β KO mice induced by STZ. They observed a significant increase in the number of metabolites with diabetes-related changes in WT mice, while those related to meprin β deficiency were decreased in plasma^93^. Given that meprin can be expressed in monocytes and macrophages, future studies can further investigate its effects on macrophages in DKD, potentially uncovering new treatment directions using metabolomics methods.

Building on this, combined treatment strategies targeting multiple key macrophage subtypes and related signaling pathways can be developed to enhance treatment precision. Since different drug categories target various cell types in the kidney, combined therapies may yield the strongest therapeutic effects. Currently, the combination of antihypertensive and anti-diabetic treatments is the most common and effective approach. In the future, targeting macrophages in conjunction with antihypertensive and anti-diabetic treatments may present a novel direction for treating DKD. For instance, researchers employed RNA sequencing to analyze the responses of five treatment regimens in db/db mouse models: control, ACEi, rosiglitazone, SGLT2i, ACEi + rosiglitazone, and ACEi + SGLT2i, specifically focusing on different renal cells, particularly macrophages. They discovered that combined treatments of ACEi plus rosiglitazone or ACEi plus SGLT2i had the most substantial effects^94^.

Furthermore, it is feasible to design drug carriers capable of overcoming physiological barriers and achieving precise targeted delivery. For instance, nanoparticles can be engineered using nanotechnology and surface-modified to specifically recognize and bind to markers on the surface of macrophages in kidney lesion areas, thereby enabling efficient drug delivery. Additionally, the sample size of clinical trials should be increased, and stringent inclusion and exclusion criteria should be established to ensure the representativeness of the research subjects. The observation period should also be extended to comprehensively evaluate the long-term efficacy and safety of the drugs.

## Conclusion and Outlook

The primary management of DKD currently focuses on controlling blood glucose and blood pressure, highlighting the urgent need for more effective treatments to halt the progression of kidney damage. Despite advancements in DKD therapy, a detailed characterization of macrophages and their phenotypic changes in DKD remains lacking. Macrophages-targeted therapies face significant challenges. Existing treatments are essential but are limited by considerable long-term side effects. Emerging biologics show potential but require further research and validation. Macrophage plasticity and metabolism are untapped the key message therapeutic levers in DKD. Future research should prioritize the development of precise and personalized treatment strategies to improve patient outcomes.

The application of new omics technologies, such as single-nucleus RNA sequencing, phosphoproteomics, and metabolomics, can provide deeper insights into the pathogenesis of DKD. These technologies will elucidate the molecular underpinnings of the disease, reveal pathogenic mechanisms, and identify potential therapeutic targets, facilitating personalized treatment plans that promise greater efficacy and reduced side effects. Integrating traditional Chinese and Western medicine will be a key research focus, effectively combining these two systems.

In conclusion, a comprehensive understanding of the role of macrophages in DKD will aid in identifying novel therapeutic targets. Macrophages involved in inflammation and fibrosis offer promising targets for developing innovative DKD treatment strategies.

## Abbreviations

DKD: Diabetic kidney disease
ESRD: end-stage renal disease
DM: Diabetes Mellitus
CKD: chronic kidney disease
ADA: American Diabetes Association
KidneyDisease: Improving Global Outcomes (KDIGO)
eGFR: estimated glomerular filtration rate
ACR: albumin-to-creatinine ratio
ROS: reactive oxygen species
HG: high glucose
TNF: tumor necrosis factor
APCs: antigen-presenting cells
MMT: macrophage-myofibroblast transition
LN: lupus nephritis
Ics: immune complex deposition
IgAN: IgA nephropathy
NLRP3: Nucleotide binding oligomerization domain-like pyrin domain-containing protein 3
PMT: podocyte-macrophage transdifferentiation
CASK: calcium/calmodulin-dependent serine/threonine kinase
FSGS: focal segmental glomerulosclerosis
MNP: mononuclear phagocyte
PI3K: phosphatidylinositol 3-kinase
AKT: protein kinase B
NF-κB: Nuclear Factor kappa-light-chain-enhancer of activated B cells
RAC1: Ras-related C3 botulinum toxin substrate 1
IFN-γ: interferon-gamma
LPS: lipopolysaccharides 1
CRP: C-reactive protein
MHC II: major histocompatibility complex II
IL-1R: interleukin-1 receptor
HLA-DR: human leukocyte antigen-DR
Nrf2: nuclear factor-erythroid 2 related factor 2
KO: knockout
MRP8: myeloid-related protein 8
AP-1: protein 1
CEBP/β: CCAAT enhancer binding protein beta
JAK-STAT: Janus kinase-signal transducer and activators of transcription
OPN: osteopontin
TGF-β: transforming growth factor-β
ERK: extracellular signal-regulated protein kinases
MAPK: mitogen-activated protein kinase
JNK: Jun N-terminal kinase
MSCs: mesenchymal stromal cells
TFEB: transcription factor EB
STZ: streptozotocin
HK-2: renal tubular epithelial cell
EVs: extracellular vesicles
RT-qPCR: Reverse Transcription-quantitative Polymerase Chain Reaction
HIF-1α: Hypoxia-inducible factor-1α
Chk2: checkpoint kinase 2
BCAA: branched-chain amino acids
BCKA: branched-chain keto acids
NO: nitric oxide
MIP-1β: macrophage inflammatory protein-1β
RasGRP4: Ras guanine nucleotide-releasing protein-4
ICAM-1: intercellular cell adhesion molecule-1
VCAM-1: vascular cell adhesion molecule-1
PBMCs: peripheral blood mononuclear cells
lncRNA: long non-coding RNA
PPARγ: peroxisome proliferator-activated receptor γ
NGAL: neutrophil gelatinase-associated lipocalin
MCP-1: Monocyte chemoattractant protein-1
CCL2: Chemokine (C-C motif) ligand 2
CUL4B: Cullin4B
ITGA9: increasing integrin α9
ECM: extracellular matrix
EMT: epithelial-mesenchymal transition
EndoMT: endothelial-mesenchymal transition
FGF-2: fibroblast growth factor 2
PDGF: platelet-derived growth factor
UACR: urinary albumin-creatinine ratio
FN1: Fibronectin 1
TGFBI: transforming growth factor beta-induced
GLP-1RAs: glucagon-like peptide-1 receptor agonists
SGLT2i: sodium-glucose cotransporter-2 inhibitors
DPP-4: dipeptidyl peptidase-4
RAAS: renin-angiotensin-aldosterone system
ACEi: angiotensin-converting enzyme inhibitors
RPTECs: proximal tubule epithelial cells
MDMs: monocyte-derived macrophages
Cx43: connexin 43
MSC-sEV: mesenchymal stem cell-derived extracellular vesicles
CK1δ: casein kinase 1 delta
β-TRCP: β-transducin repeat-containing protein
YAP: Yes-Associated Protein
GEO: Gene Expression Omnibus
WGCNA: Weighted Gene Co-expression Network Analysis
TCM: raditional Chinese medicine
HPS: Hyperoside
FACS: fluorescence activated cell sorting
WAT: white adipose tissue
DIO: diet-induced obese
SD: Sprague-Dawley
NC: normal control
TREM-1: Triggering Receptor Expressed on Myeloid Cells 1
PRDX2: peroxiredoxin 2
TLR4: toll-like receptor 4
